# Towards Performant Design of Carbon-Based Nanomotors for Hydrogen Separation through Molecular Dynamics Simulations

**DOI:** 10.3390/ijms21249588

**Published:** 2020-12-16

**Authors:** Sebastian Muraru, Mariana Ionita

**Affiliations:** 1 Faculty of Medical Engineering, University Politehnica of Bucharest, GhPolizu 1-7, 011061 Bucharest, Romania; sebmuraru@gmail.com; 2 Advanced Polymer Materials Group, University Politehnica of Bucharest, GhPolizu 1-7, 011061 Bucharest, Romania

**Keywords:** hydrogen separation, rotating carbon nanotube membrane, molecular dynamics

## Abstract

Clean energy technologies represent a hot topic for research communities worldwide. Hydrogen fuel, a prized alternative to fossil fuels, displays weaknesses such as the poisoning by impurities of the precious metal catalyst which controls the reaction involved in its production. Thus, separating H_2_ out of the other gases, meaning CH_4_, CO, CO_2_, N_2_, and H_2_O is essential. We present a rotating partially double-walled carbon nanotube membrane design for hydrogen separation and evaluate its performance using molecular dynamics simulations by imposing three discrete angular velocities. We provide a nano-perspective of the gas behaviors inside the membrane and extract key insights from the filtration process, pore placement, flux, and permeance of the membrane. We display a very high selectivity case (ω = 180° ps^−1^) and show that the outcome of Molecular Dynamics (MD) simulations can be both intuitive and counter-intuitive when increasing the ω parameter (ω = 270° ps^−1^; ω = 360° ps^−1^). Thus, in the highly selective, ω = 180° ps^−1^, only H_2_ molecules and 1–2 H_2_O molecules pass into the filtrate area. In the ω = 270° ps^−1^, H_2_, CO, CH_4_, N_2_, and H_2_O molecules were observed to pass, while, perhaps counter-intuitively, in the third case, with the highest imposed angular velocity of 360° ps^−1^ only CH_4_ and H_2_ molecules were able to pass through the pores leading to the filtrate area.

## 1. Introduction

Predominantly, hydrogen meant to be used as fuel is obtained through steam reforming gas, with the following chemical reactions involved [CH_4_ + H_2_O → CO + 3H_2_; CO + H_2_O → CO_2_ + H_2_]. Any impurities, such as CH_4_, CO_2_, CO, N_2_, and H_2_O molecules can significantly reduce the performance of a hydrogen fuel cell by poisoning its precious metal catalyst driving forward the electrochemical reactions [1,2]. Thus, separating the H_2_ molecules out of the mixture of gases is crucial for keeping fuel cells efficient. Mature separation technologies such as pressure swing absorption and cryogenic distillation have lost ground to newer solutions, e.g., separation membranes due to their affordability and easy operation [2,3,4,5]. Many of these have been designed using different metals, silica, zeolite, various polymers, and carbon, yet without coming upon an ideal solution [1,3,4,5,6,7].

Carbon, as one of the most common and versatile elements in the universe, has continuously attracted significant interest within the scientific community over the span of several decades. Constant discovery of new allotropes, each displaying unique properties, allowed for carbon to be used for an extraordinary array of purposes and applications [8,9]. In particular, synthetic allotropes such as the one-dimensional carbon nanotubes [10] and the two-dimensional graphene [11] have ignited enormous interest within the materials science community. Despite being impermeable to water and gas molecules, techniques such as ion bombardment or e-beam lithography [3,12] can be used to create tailored nano-pores and permeabilize these structures.

Molecular dynamics (MD) simulations represent a computational technique useful in acquiring a nano, atom-level perspective of all molecules making up a defined system. Complete trajectories are built step-by-step for each atom present in a simulation box. Although it is used ultimately to complement experimental research, it may also be used to uncover key insights entirely on its own. This is possible because a computational researcher can explore scenarios that may prove too costly, time-consuming, or simply inaccessible yet to experimental research. Here, we make use of MD simulations to explore certain useful parameters during hydrogen separation using a theoretical framework. Several MD-only studies have investigated the molecular mechanisms useful in describing the separation potential of carbon-based structures, either for gas separation [12,13,14,15,16,17,18] or water desalination [19,20].

The recently observed rotational motion of double-walled carbon nanotubes exposed to an electric field [21] opened the door to more complex MD simulations of carbon-based nanomotors, investigating areas such as behavior in water, water transport, and desalination [21,22,23,24]. To our knowledge, there are no existing MD studies on carbon-based nanomotors evaluating gas separation. In this study, we explored the impact of an imposed angular velocity on the performance of H_2_ separation of a rotating partially double-walled carbon nanotube design out of a mixture of gases containing H_2_, CH_4_, CO, CO_2_, N_2_, and H_2_O molecules. The gases, placed inside the rotating carbon nanotube could either exit through the nanopores present along the length of the nanotube or through one of its ends which was left open. Performance was determined by analyzing the filtrated molecules, pore placement, and exploring parameters such as flux and permeance. In a practical setting, the angular velocity imposed on the rotating carbon nanotube can be generated by the presence of an electric field, either directly or through a gear-based mechanism, for which we have provided a segment on our design, leaving it pore-free. We found a favorable case of imposed angular velocity leading to impressive selectivity (ω = 180° ps^−1^) and showed that the outcome of MD simulations can be both intuitive and counter-intuitive when increasing the ω parameter (ω = 270° ps^−1^; ω = 360° ps^−1^). The design allows for the gas mixture to be exposed to a high surface area; each gas molecule always traveling towards a pore. H_2_ molecules were able to quickly exit the rotating nanotube by the 10ns mark and no gas molecules were able to cross back into the membrane leading to overall increased efficiency. In addition, flux values were comparable to graphene pores corresponding to 10-16 removed atoms. Overall, we have shown that the angular velocity plays a significant role in hydrogen separation through a rotating nanotube and discussed key parameters involved in the separation process.

## 2. Results and Discussion

### 2.1. Choosing Pore Shape and Size

**Figure 1:**
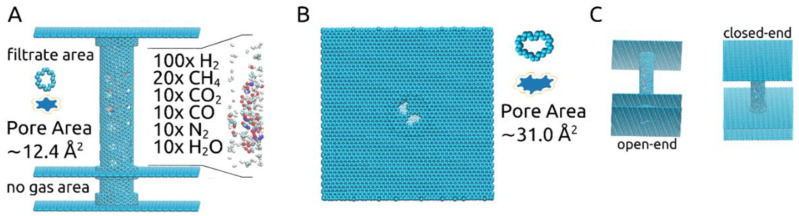
(**A**) Outline of the rotating carbon nanotube membrane, together with gas mixture and small pore details; (**B**) View of the open-end together with the larger pore details; (**C**) Perspectives on the membrane showing the open-end and the closed-end.

In this study, the pores’ shapes and sizes, shown in Figure 1A,B, were chosen based on our previous investigation on gas separation membranes [18]. The larger pore, corresponding to the removal of 14-atoms out of a graphene sheet, was proven to allow all molecules of the described gas mixture to pass through, while the smaller pore, corresponding to the removal of a 6-atom graphene ring, was shown to allow only the passage of H_2_ and H_2_O molecules.

**Figure 2:**
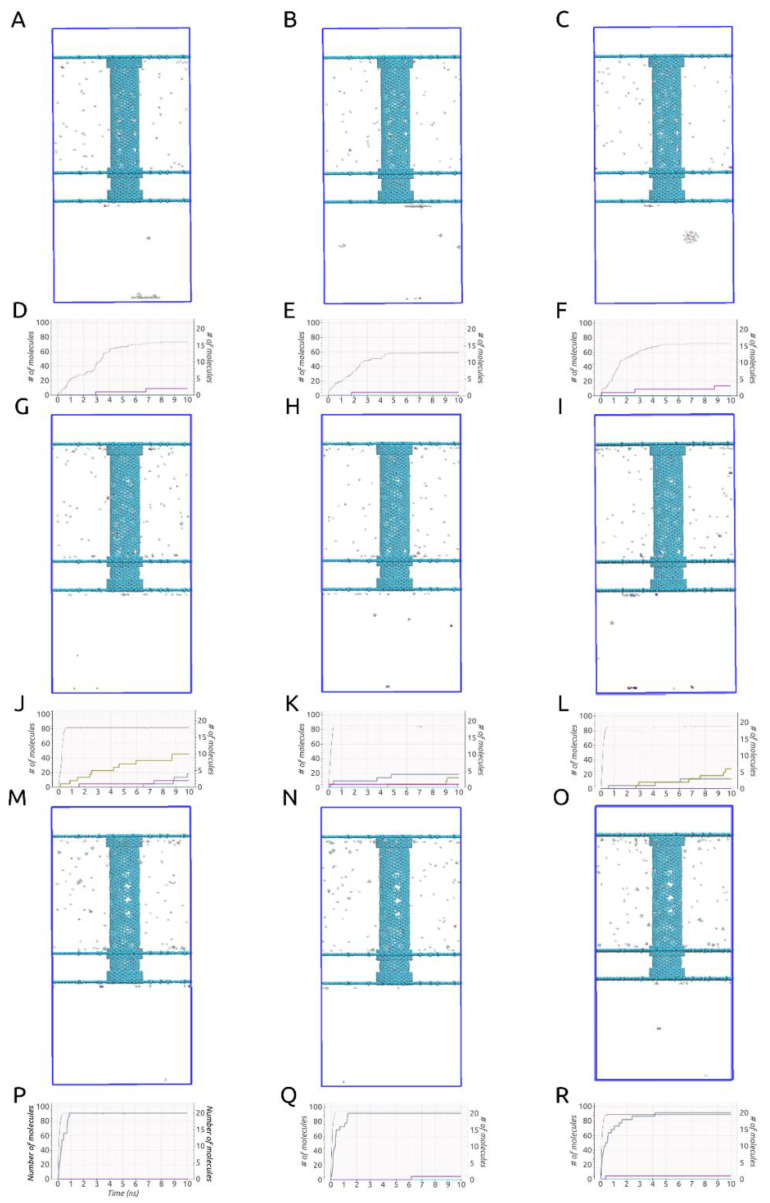
(**A**–**C**). (ω = 180° ps^−1^), (**G**–**I**). (ω = 270° ps^−1^), (**M**–**O**). (ω = 360° ps^−1^) Final frame of the MD simulations (10 ns); (**D**–**F**). (ω = 180° ps^−1^), (**J**–**L**). (ω = 270° ps^−1^), (**P**–**R**). (ω = 360° ps^−1^) Evolution of the number of molecules passing into the filtrate area during simulations time.

**Figure 3:**
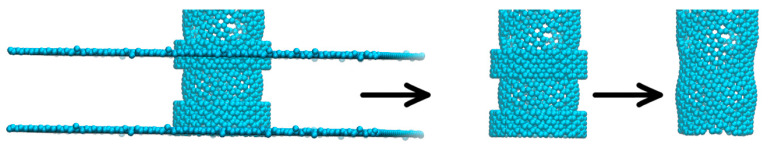
Example of the structural deformation occurring in the rotating carbon nanotube due to imposed angular velocity.

An important aspect to mention when conducting investigations similar to ours, obvious when looking at Figure 2A–C,G–I,M–O, is that some deformation of the carbon nanotube occurred during each of the simulations due to the imposed rotational movements. This aspect can be observed easiest by looking at the no gas area. The same area is specifically shown in Figure 3. These structural deformations can lead to changes that occur in the shape and size of the pores situated on the rotating carbon nanotube and thus different gas separation behaviors. In addition, looking at Figure 2D–F,J–L,P–R, no gas molecules ever cross back into the membrane. The small fluctuations seen in the case of H_2_ molecules are due to the calculation method (the H_2_ number on the graphs always goes back up quickly).

Effective pore areas A_p_ were determined using a Monte Carlo hit-and-miss procedure [14,16,18], considering the effective carbon atom radius R_eff_ = R_m,c_/√2 and R_m,c_ equal to 0.17 nm. The approximate results are shown numerically in Figure 1A,B, meaning ~12.4Å^2^ for the smaller pores located along the carbon nanotube and ~31.0Å^2^ for the larger pores placed in the graphene layer at one of its ends. Blue-filled shapes for each pore type showing their effective pore areas are also shown in Figure 1A,B (images not to scale). The two values were calculated considering the pores to be sculpted in a planar 2D graphene sheet. This approach fits very well in the case of the larger pore. For the smaller pore, however, determining the area in such a manner is less accurate due to being sculpted in a carbon nanotube with a chiral vector determined by the indices (12, 16). Nevertheless, it still provides a useful approximate value. Using the same method for calculating the smaller pore’s area, now when sculpted in the chiral nanotube, we ended up with the following approximate values: 12.26 Å^2^ at 0ns before the start of the simulations, 12.51Å^2^ for ω = 180° ps^−1^, 13.02Å^2^ for ω = 270° ps^−1^ and 14.70Å^2^ for ω = 360° ps^−1^.

Thus, given the imposed angular velocities to the carbon nanotube, changes in the shape and size of the smaller pores took place. The shape and size of the pores, together with the angular velocity of the nanotube are the main parameters behind the distinct results obtained here for each ω case. This aspect helps explain the results obtained in this paper. The influence of an imposed angular velocity on the geometry of a carbon nanotube and pores sculpted on its surface can be investigated in future studies.

The rationale behind keeping an “open” end to the membrane, represented by the graphene layer with the large pores placed as pictured in Figure 1A,B, was to allow for the gas mixture to be fed into the carbon nanotube. This obviously led to some H_2_ molecules to be lost as they were able to exit the membrane through the pores situated at the end of the tube instead of crossing through the smaller pores into the filtrate area. However, we deemed this “open” end setup more realistic and practical. Given our previous results [18], which confirmed that all gas molecules present in the gas mixture used here are able to pass through the 31.0 Å^2^ pore, we focused the current investigation on the separation process that would occur once the gas mixture is already loaded into the rotating carbon nanotube membrane and the effect of the imposed angular velocity.

### 2.2. Simulation Results

Curiously, given the setups described, all three ω cases yielded significantly different results, consistent throughout all three runs, as can be observed in Figure 2. We must mention that in all nine simulations, all H_2_ molecules had either crossed through the smaller pores or the larger pores by the 10 ns mark. Thus, all of them had left the rotating carbon nanotube by the time-point at which we stopped our simulations. On top of that, given the three tested values for the imposed angular velocity, at least 60% of the H_2_ molecules were able to cross into the filtrate area within the simulated time in all given setups.

### 2.3. Angular Velocity ω = 180° ps^−1^

In the case of ω = 180° ps^−1^, consistent with our previous investigations [18], only H_2_ and H_2_O molecules were able to pass through the smaller pores. The process of H_2_ molecules passing through the smaller pores and entering the filtrate area took place in about 7–8 ns, with ~20 H_2_ molecules passing within the first nanosecond and some other 40 to 50 molecules within the next 6–7 ns. In regards to H_2_O molecules, only 1–2 molecules were able to pass into the filtrate area, while most of them (6–7) were still found inside the carbon nanotube at the end of the 10 ns. The reason for this is that although the smaller pores should theoretically allow them to pass through, these are found to cluster together inside the nanotube due to hydrogen bonding. In all our simulations this prevents most of them from leaving the rotating membrane, given the pore sizes used. No other gas molecules were able to pass through the smaller pores at this angular velocity.

**Figure 4:**
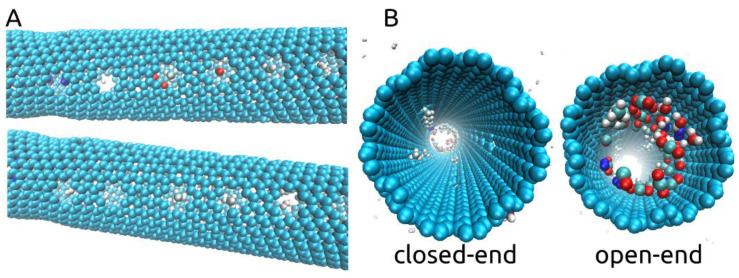
(**A**). Cases of N_2_, CH_4_, CO, and H_2_ gas molecules shown to block some of the pores situated along the rotating central carbon nanotube; (**B**). Closed-end and open-end views at the end of the simulations (ω = 180° ps^−1^) showing the agglomeration of the molecules at one of the ends and the CH_4_ molecules still blocking some of the pores.

Taking a look at the process of separation occurring throughout our simulations, we observed that some of the gas molecules inside the nanotube position themselves as if blocking the smaller pores and remain positioned so, despite the rotating motion. This aspect is shown in Supplementary Video S1 and in Figure 4A with CH_4_, CO, H_2_O, N_2_, and H_2_ molecules blocking some of the pores. We also show, in Figure 4B, that by the end of the simulation, at 10ns, pores were still blocked by the larger molecules at a time at which H_2_ molecules were no longer found inside the rotating nanotube. We think that due to the imposed rotational motion and the size and shape of the chosen smaller pores, the smaller molecules such as H_2_ and H_2_O are able to eventually exit through the pores they are blocking. Thus, with an imposed ω of 180° ps^−1^, some degree of fouling will occur, leaving fewer pores open for H_2_ molecules to cross and leading more to pass through the open end of the membrane in exchange for the high selectivity offered. Between 59 and 73 out of 100 H_2_ molecules were able to pass into the filtrate area throughout the three simulations with an imposed angular velocity of 180° ps^−1^. Interestingly, no CO_2_ molecules were found to block any of the smaller pores throughout all simulations but were instead able to pass through the larger pores as can be seen in Figure 2I.

At ω = 180° ps^−1^, as it can be observed in Supplementary Video S1, some H_2_ adsorption to the wall of the rotating carbon nanotube took place, which allowed the gas molecules to travel with the nanotube in its rotating motion. However, the phenomenon lasted for a short time (a few ps) before the adsorbed molecules were disturbed. We think both the imposed high angular velocity and the placement of the pores along the membrane, with each pore crossing disturbing the nearby gas molecules, help prevent adsorption from taking place and thus allow all H_2_ molecules to leave the nanotube by the end of the simulation.

**Figure 5:**
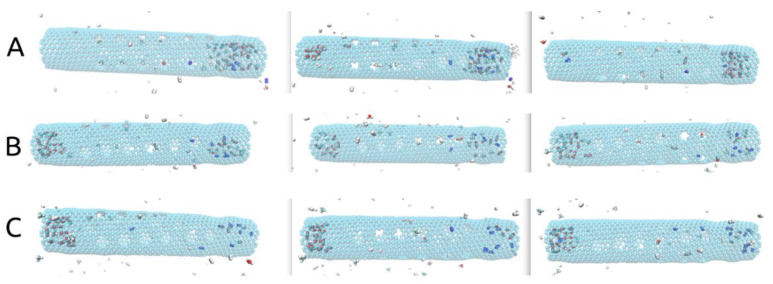
(**A**) (ω = 180° ps^−1^), (**B**) (ω = 270° ps^−1^), (**C**) (ω = 360° ps^−1^) Representations of the rotating central carbon nanotube at 10ns simulation time showing the gas molecules gathered at one of its ends.

Another important aspect to discuss in the presented rotating carbon nanotube membrane design regards the placement of the pores leading to the filtrate area along the tube. In our case, as shown in Figure 1, we have placed the 24 smaller pores in the central part of the nanotube, leaving both ends with no pores to the filtrate area. As shown in Figure 5, at the end of all simulations (10 ns), unless found blocking one of the smaller pores, all molecules that remained in the rotating nanotube can be seen gathered at one of its ends. Without smaller pores nearby, the agglomeration of molecules becomes somewhat “stagnant” despite the rotational movement of the tube (see Supplementary Video S2). This aspect may be beneficial to the flux of H_2_ to the filtrate area as the smaller pores are then less likely to be blocked.

Additionally, inspecting Figure 5, all H_2_ molecules exited by the 10ns mark, and only CH_4_, CO, CO_2_, N_2_, and H_2_O molecules (clustered) can be seen inside. To provide an explanation for this we looked at the movement of H_2_ molecules.

As shown in Supplementary Video S2, the molecules in the mixture with a smaller mass, meaning H_2_, CH_4_, and H_2_O were able to move slightly quicker when inside the rotating nanotube. On top of that, due to their small volume, which allowed them to “squeeze” through the larger molecules and their tendency to adsorb to the wall of the nanotube, H_2_ molecules had a high number of collisions. Together with the chirality of the nanotube, we think these aspects lead to the exiting of all H_2_ molecules within the 10 ns timeframe as they are more likely to travel along the nanotube and exit either towards the filtrate area or through the “open” end. The movement of H_2_ molecules within rotating carbon nanotubes of different chiralities could be investigated in future studies to determine whether chirality can drive the gas molecules due to their tendency to adsorb to the wall of the nanotube.

### 2.4. Angular Velocity ω = 270° ps^−1^

For the higher imposed angular velocities, significantly different outcomes were observed compared to the ω = 180° ps^−1^ case. The outcomes were consistent throughout all three repetitions corresponding to a certain ω value. In the case of ω = 270° ps^−1^, H_2_, CO, CH_4_, N_2_, and H_2_O molecules were observed to pass through the smaller pores, as shown in Figure 2G–L. Almost all H_2_ molecules that passed into the filtrate area (~80–85) did so in the first half nanosecond. Again, no H_2_ molecules were found inside the tube at the end of the 10 ns. Most molecules that passed through the smaller pores did so after blocking the pore for a while and exited either due to a collision with another gas molecule or due to the rotational motion of the nanotube, which allowed for slight changes in the orientation of the molecules. At this imposed angular velocity, despite that more H_2_ molecules reached the filtrate area, there was far less selectivity leading to a poor separation performance.

The movement of the H_2_ molecules inside the tube was slightly different compared to the ω = 180° ps^−1^ case, please see Supplementary Video S3. Due to the higher imposed angular velocity, H_2_ molecules that were not blocking a pore were far less able to adsorb to the wall of the tube and travel in a synchronized manner with its rotational motion (as observed when ω = 180° ps^−1^). Thus, due to the collisions with the wall, some H_2_ molecules traveling in a rotational motion nearby, did so both in the sense of the rotation and in the anti-sense, but slower when compared to the rotating wall. Essentially, this should have led to a higher number of collisions taking place inside the tube, which, alongside with the enlargement of the pores due to the larger angular velocity, enabled most molecules to exit into the filtrate area. CH_4_ molecules were also observed to pass through the pores after blocking it for a while and following a collision. No CO_2_ molecules were able to pass through the smaller pores. The results obtained for ω = 270° ps^−1^ case are somewhat intuitive in that the higher angular velocity led to the expansion of the smaller pores and plummeted its selectivity while allowing more H_2_ molecules to pass into the filtrate area. From these simulations one can also see the following insight: as most remaining gas molecules at the 10ns mark are either found blocking one of the smaller pores or situated at one of the ends of the tube, the placement of the smaller pores along the center of the rotating carbon nanotube membrane could limit the passage of larger gas molecules into the filtrate area, thus improving the performance of the membrane.

### 2.5. Angular Velocity ω = 360° ps^−1^

In the final case of ω = 360° ps^−1^, as shown in Figure 2M–R, 89 to 94 H_2_ molecules were able to pass through the smaller pores in less than 0.5 ns. The H_2_ molecules that passed through the larger pores did so within 0.3 ns since the start of the simulations. Curiously, the only other gases which passed into the filtrate area at the end of the simulated time were CH_4_ and H_2_O molecules. Consistent throughout all three simulations with ω = 360° ps^−1^, all 20 CH_4_ molecules passed through the smaller pores and, similarly to the previously mentioned cases, ω = 180° ps^−1^ and ω = 270° ps^−1^, only one H_2_O molecule managed to do so, due to the clustering of the water molecules inside the tube. The fact that these results were consistent throughout all three repetitions shows, counter-intuitively, that using higher angular velocities values can lead to unique and unexpected insights. The fact that almost 90–95% of H_2_ molecules placed initially in the tube, together with 100% of the CH_4_ molecules and only 10% of the water molecules were able to pass into the filtrate area could be useful in future studies working on improving the current design by making use of a gradual separative process. In such a case, the second step would involve only the separation of H_2_ and CH_4_ molecules, as, curiously, no other gas molecules were able to pass into the filtrate area.

Observing the movement of the molecules inside the rotating carbon nanotube at ω = 360° ps^−1^ shows the molecules with a smaller mass, H_2_, CH_4_, and H_2_O moving significantly faster than the larger CO, N_2_, and CO_2_ molecules (see Supplementary Video S4). On top of that, due to collisions with the walls of the tube, the same smaller mass molecules rotate around their own center of mass much quicker than the larger ones, with CH_4_ being a special case and rotating faster than all linear molecules due to its geometry. Further on, given the quick rotation of the tube, no gas molecules were seen blocking any of the smaller pores or being able to adsorb to the rotating walls of the membrane. Thus, we think that when a gas molecule reaches the vicinity of a pore, due to the high angular velocity of the nanotube, the right orientation has to be found quickly in order to exit, which did not happen in the case of the larger linear molecules with a higher mass, but did take place in the case of the non-linear molecules with a smaller mass, meaning CH_4_ and H_2_O molecules. That is, gas molecules had a very short time to match an orientation that would have allowed them to cross the pore. After nearing a pore and colliding with the rotating wall, a small mass non-linear molecule was more likely to quickly rotate around its mass center and change its orientation quickly, which increased its chances of crossing. However, despite its linear structure, H_2_ molecules crossed the smaller pores easily due to their very small mass and volume as these aspects allowed them to change their orientation quickly and match that necessary to exit through the pore. More details, solely in regards to pore crossing can be reached in further investigations through ab initio molecular dynamics (AIMD) techniques.

### 2.6. Flux and Permeance

**Figure 6:**
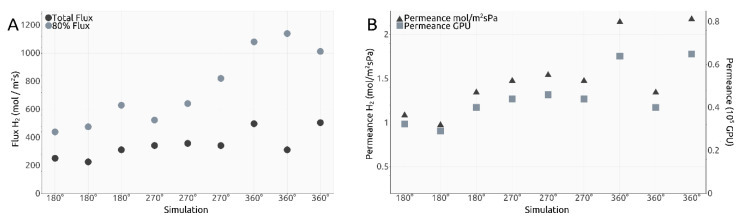
(**A**). Calculated total flux and 80% flux values; (**B**). Calculated permeance values in mol m^−2^ s^−1^ Pa^−1^ and Gas Permeation Units (GPU).

To further characterize the rotating carbon nanotube design for gas separation, we calculated the flux of H_2_ molecules through the smaller pores. Thus, in order to determine the total flux we counted the number of crossings from both inside and outside the tube and then made use of the formula below:

(1) $$Flux=\frac{Crossing\;s_{H2}/N_{A}}{A_{innersurface}\times Time}$$

where *N_A_* represents Avogadro’s number, *A_inner_surface_* represents the inner surface of the tube and time corresponds to the time point of the last H_2_ crossing during the simulation. In addition, given that the majority of H_2_ molecules exit in a time window significantly shorter than the duration of the whole simulation, we have calculated the flux within the time frame in which 80% of H_2_ molecules had left the rotating carbon nanotube membrane. The inner surface of the tube was determined to be 59.854 nm^2^ using the formula 2πrh. The radius of the tube, r, was determined using the formula:

(2) $$Radius=\frac{a}{2\pi }\times \surd \left(m\times m+m\times n+n\times n\right)$$

where a = 0.246 nm and m and n represent the chiral indices of the nanotube. The results are shown in Figure 6A. Given the calculated total flux values, permeance values for our membrane design were estimated and shown both in mol/m^2^sPa and gas permeation units (GPUs) in Figure 6B.

Dividing the flux values presented in Figure 6A to the number of smaller pores present along the inner surface of the carbon nanotube, we obtain the intervals shown in Table 1:

Despite the very different setups, comparing the total flux values obtained here (shown in Table 1) after division to the number of relevant pores, with the values presented in our previous research for a different design with the same gas separation purpose, the results are not significantly different from the interval 4.5–14.0 mol/m^2^s observed in [18] on the third filtration layer. The 80% flux intervals presented in Table 1 are higher, yet that is unsurprising, but these importantly highlight the manner in which the H_2_ molecules cross the smaller pores in the current design, with most of them managing to exit the rotating nanotube in the first 5.0 ns or 0.5 ns depending on the ω case, yet much quicker than the last 20%.

Previous results were exceptional in terms of selectivity and permeability due to the gradual separation through multiple graphene layers each with its own specific pore size [18]. In the current design, we observed different behaviors, yet the exit of all H_2_ molecules took place in a very short time and no H_2_ molecules were left inside the nanotube at the end of the simulated 10 ns. Thus, nanopores used for filtration can be customized for certain molecules not only by selecting their size and 2D shape but also by using an imposed angular velocity and the forces involved in the consequent rotational motion, thus changing its geometry. Therefore, using a rotating carbon nanotube membrane allowed the gas molecules to be exposed to most of the surface area of the membrane, uncovering interesting distinct behaviors, dependent on the imposed angular velocity. Nevertheless, alongside the advantage of the quick exiting of all H_2_ molecules within the membrane, a disadvantage of the current design is the loss of some of them due to the “open” end. Future studies may investigate different scenarios for improving the efficiency of the membrane. The easiest scenario to imagine being blocking the open end with a full graphene layer with no pores.

Comparing to Sun’s results [16], the total flux values obtained in Figure 6A are comparable to a graphene pore size of 10 to 12 atoms, while the 80% flux values are comparable to a 12-atom graphene pore for ω = 180° ps^−1^, 14-atom graphene pore for 270° ps^−1^ and 16-atom graphene pore for 360° ps^−1^.

Naively comparing the flux values obtained for each ω case to each other, a clear trend can be seen showing that a higher imposed angular velocity leads to generally higher flux. However, an outlier can be seen in Figure 6A, with one total flux value in the ω = 360° ps^−1^ significantly smaller than the other two. This was also one of the reasons we decided to calculate the 80% flux values. The corresponding value to the outlier for the 80% flux is higher than the two other similar simulations, which shows that most of the H_2_ molecules in the outlier simulation crossed into the filtrate area quicker and left the other 20% needing more time to cross due to, potentially, fewer collisions between molecules. Thus, with this explanation, we deem the outlier valid. One must take into account, however, that the heights of the total flux and the 80% flux values are not perfectly proportional due to events such as pore blocking, exit of other gas molecules, etc.

Similar to flux calculations, we estimated permeance values for the rotating carbon nanotube membrane, as shown in Figure 6B. Similar conclusions can be taken out of the permeance values as with the total flux calculations. We find all our simulations to indicate a value within the following intervals: 0.0097 to 0.0215 mol^−3^/m^2^sPa and 0.30 to 0.65 × 10^5^ GPU, and thus in the vicinity of those calculated for our previous design, with values situated between 0.005 to 0.03 mol^−3^/m^2^sPa and 0.20 to 0.85 × 10^5^ GPU, results already highlighted as superior to state-of-the-art solutions [18].

## 3. Materials and Methods

### 3.1. Membrane Prototype

The structure of the membrane is shown in Figure 1A,B: a central carbon nanotube with indices (12, 16) and 10 nm in length is held in place by two 10 nm × 10 nm graphene sheets placed at its ends. At one end the graphene sheet is intact (“closed-end”), while at the other, two pores corresponding to the removal of 14-atoms were made (“open-end”). The central carbon nanotube is partially doubled by three short carbon nanotubes with the indices (19, 19) and 0.5 nm in length. The radius difference corresponds to the 0.334 nm distance met in graphite sheets, situated closely, at 0.3358 nm. Two of the short carbon nanotubes are placed at the central nanotube’s ends and one is placed 2 nm away from its open end, which is then inserted in an additional 10 nm × 10 nm graphene layer, dividing the volume outside the central carbon nanotube into the “filtrate area” and “no gas area”. The nanotubes and graphene sheets were generated using VMD [25]. To make the design more applicable to experimental settings, the “no gas area” represents a 2 nm-long pore-free segment for moving the central carbon nanotube through nano gear-based mechanisms. Such may be a very complex undertaking for molecular dynamics studies due to the need of obtaining a constant rotating motion and constant velocity and thus remains to be investigated in future studies. Along the center of the carbon nanotube, four columns of six smaller pores, corresponding to the removal of six atoms making up a hexagonal ring, are present. A gas mixture of 100× H_2_, 20× CH_4_, 10× CO_2_, 10× CO, 10× N_2_, and 10× H_2_O molecules are placed randomly inside the central carbon nanotube, which is free to rotate around its own axis.

### 3.2. Simulation Details and Force Field Parameters

All simulations were run using the OPLS-AA force field, together with GROMACS 2018.3. The rotational motion of the central carbon nanotube was achieved using the Enforced Rotation module and iso-pf potential. Three different angular velocities were imposed, with ω = 180° ps^−1^, 270° ps^−1^, and 360° ps^−1^. For each case, three repetitions were run using the rotational force constant 500 kJ mol^−1^ nm^−2^. All simulations were run for 20,000,000 steps, using a step size of 0.5 fs and summing up to 10 ns simulated time. Coordinates for visualizations were saved every 5 fs. Simulation box size was 10 nm × 10 nm × 20 nm and contained ~15,000 atoms. Periodic boundary conditions were active in the X and Y directions. On the *Z*-axis, the top and bottom of the simulation box contained two walls made out of Kr atoms (Lenard Jones (LJ) 10–4) with a number density of 5 nm^−2^. In order to confer a repulsive character, interactions with the Kr atoms were cut at 0.1 nm. VdW and Coulomb cutoff was set at 1.0 nm. The water model used was SPC/E. The Verlet cut-off scheme was used, together with the V-rescale thermostat at 300 K.

Most parameters for the gas molecules were taken from previous studies using the OPLS-AA forcefield [12]. H_2_ and N_2_ molecules were modeled as three-site models, with one virtual mass-less atom in the center of the molecules, while CO_2_ molecules were built as a five-site model, thus using two virtual atoms, as shown in Lemkul’s tutorial [26]. CH_4_ and CO molecules were built without the aid of virtual atoms and thus contained five, respectively two atoms.

Carbon atoms making up the setup of the membrane were considered uncharged LJ spheres with cross-section 0.34 nm, potential wall depth 0.36 kJ mol^−1^, C-C bond length 1.42 Å, C-C-C bending angle 120°, and C-C-C-C planar angles maintained by harmonic potentials with springs constants of 322.55 kcal mol^−1^ Å^−2^, 53.35 kcal mol^−1^ rad^−2^ and 3.15 kcal mol^−1^ [27].

For data analysis we made use of Python libraries such as NumPy, bokeh [28] and MDAnalysis [29,30].

## 4. Conclusions

We have presented a rotational partial double-walled carbon nanotube hydrogen separation membrane design relying on the recently observed rotational motion of double-walled carbon nanotubes when exposed to an electric field [21] and investigated its gas separation performance under three different imposed angular velocities (180° ps^−1^, 270° ps^−1^, and 360° ps^−1^), while observing the movement of the gases inside the membrane. We have shown that the outcomes of the simulations are dependent on the structural changes occurring in the rotating membrane and affecting the size and shape of the smaller pores leading to the filtrate area. We have presented a case of very high selectivity for the current setup (ω = 180° ps^−1^) and highlighted the advantages of the design such as the fast exit of the H_2_ molecules from within the nanotube. We have provided explanations for its interesting strong points such as the exit of all H_2_ molecules within simulated time irrespective of the behavior of the other gases, a phenomenon consistent through all our simulations. Given the significantly different simulation outcomes, we deem that in an experimental setting, tightly controlling the angular velocity would be paramount. Given the imposed angular velocity cases presented, we have shown that the outcome of MD simulations can be both intuitive and counter-intuitive: compared to the 180° ps^−1^ case, an increase in the flux of H_2_ molecules in the 270° ps^−1^ case came with a plummeting of the selectivity and allowed many other gas molecules to pass into the filtrate area; however, a further increase of the imposed angular velocity to 360° ps^−1^ lead to the exit through the smaller pores of only H_2_ and CH_4_ molecules in spite of the increased pore size, making for a very interesting insight useful in gradual separation designs. Compared to other designs, in the presented prototype, the gas mixture was exposed to a large surface area with each gas molecule always traveling towards a pore. In addition, no gas molecules were able to cross back into the membrane.

We have provided flux and permeance estimates and found them comparable to our previous designs and superior to existing experimental solutions [18]. On top of that, our 80% flux values were found comparable to 12-atoms, 14-atoms, and 16-atoms graphene pores [16]. Thus, we have shown that the angular velocity plays a significant role in hydrogen separation through a rotating nanotube. We deem further experimentation involving MD investigations on novel gas separation membrane designs will lead to valuable insights for the hydrogen fuel industry.

## Figures and Tables

**Table 1:** Resulting flux intervals after dividing flux values to the number of smaller pores.

Angular Velocity-ω (deg/ps)	Total Flux (mol/m^2^s)	80% Flux (mol/m^2^s)
180	9.0–13.0	18.0–26.0
270	14.0–15.0	21.5–34.0
360	13.0–21.0	42.0–47.5

## Supplementary Materials

The following are available online at https://www.mdpi.com/1422-0067/21/24/9588/s1.

## Abbreviations

MD	Molecular Dynamics
